# Sinomenine inhibits amyloid beta-induced astrocyte activation and protects neurons against indirect toxicity

**DOI:** 10.1186/s13041-020-00569-6

**Published:** 2020-03-04

**Authors:** Deepali Singh, Apurva Agrawal, Chitra Mohinder Singh Singal, Hriday Shanker Pandey, Pankaj Seth, Shiv Kumar Sharma

**Affiliations:** National Brain Research Centre, Manesar, 122052 Haryana India

**Keywords:** Alzheimer’s disease, Amyloid beta, Sinomenine, Neurotoxicity, Neuroinflammation

## Abstract

Amyloid beta is a major constituent of the plaques found in the brains of patients suffering from Alzheimer’s disease (AD). A growing body of research work suggests that neuroinflammation plays important roles in the development of AD. Thus, considerable efforts are directed towards identification of compounds that can reduce or inhibit neuroinflammation. Here, we show that sinomenine, a compound present in a Chinese medicinal plant, Sinomenium acutum, inhibits oligomeric amyloid beta-induced production of reactive oxygen species (ROS), nitric oxide (NO) and inflammation-related molecules from astrocytic cells. The conditioned medium from oligomeric amyloid beta-treated astrocytic cells induces cell death in the hippocampal neuronal cells. Importantly, sinomenine inhibits this cell death. In addition, this compound has inhibitory effects on the production of ROS, NO and inflammation-related factors from oligomeric amyloid-beta treated human astrocytes. Finally, the conditioned medium from oligomeric amyloid beta-treated human astrocytes induces cell death in the primary culture of human neurons, which is inhibited by sinomenine. Thus, sinomenine inhibits amyloid beta-induced production of toxic factors from astrocytes, and confers protection to hippocampal neuronal cells as well as human neurons against indirect toxicity. The results suggest that this compound could provide beneficial effects in AD and other neurodegenerative conditions by reducing inflammation and neuronal cell death.

## Introduction

Alzheimer’s disease (AD) is a neurodegenerative disorder characterized by the presence of amyloid plaques and neurofibrillary tangles. Amyloid beta, a major constituent of the plaques, is generated by proteolytic cleavage of amyloid precursor protein by beta and gamma secretases [1]. Amyloid beta aggregates into oligomers and fibrils. The oligomeric form of amyloid beta has been detected in the brains of AD patients and in the brains of animal model of AD [2, 3]. Studies have shown potent effect of amyloid beta oligomers in various processes including impairment in synaptic plasticity and memory [4].

The available literature suggests that neuroinflammation is a contributing factor in the development of neurodegenerative diseases including AD [5–8]. Thus, targeting inflammation may have a beneficial effect in AD [9]. In addition to microglia, astrocytes also contribute to neuroinflammation. Astrocytes participate in the formation of tripartite synapses and help in neuronal function. However, over-activation of astrocytes can cause neuronal damage. Amyloid beta induces reactive features in astrocytes including increase in the level of glial fibrillary acidic protein [10]. Reactive astrocytes are found in the brains of animal model of AD as well as in the brains of AD patients [9].

Considering the role of neuroinflammation in neurodegenerative diseases, significant efforts are directed towards identification of compounds, which can prevent or at least reduce neuroinflammation. Sinomenine, an alkaloid, is found in a Chinese medicinal plant, Sinomenium acutum. The chemical structure of sinomenine is provided in an article by Liu and colleagues [11]. This natural compound displays several beneficial properties [12, 13]. Qian and colleagues have shown that sinomenine inhibits lipopolysaccharide (LPS)-induced production of reactive oxygen species (ROS) from microglial cells [14]. Sinomenine protects dopaminergic neurons against neurotoxicity induced by LPS- and 1-methyl-4-phenylpyridinium in neuron-glia cultures [14]. These authors showed also that sinomenine inhibits LPS-induced production of nitric oxide (NO) and TNF-α from microglial cells. Sinomenine inhibits activation of microglia by advanced glycation end products [15]. This compound inhibits the production of inflammatory molecules and provides protection in middle cerebral artery occlusion mice model [16]. Furthermore, sinomenine reduces neurological deficits in intracerebral hemorrhage model [17].

We have previously shown that sinomenine inhibits microglial activation induced by amyloid beta, and protects neurons from indirect toxicity [18]. However, the effects of sinomenine on astrocyte activation induced by amyloid beta are not known. In the present study, we show that sinomenine prevents amyloid beta-induced increase in the levels of ROS, NO and inflammation-related molecules in the astrocytic cells. This compound protects hippocampal neuronal cells from amyloid beta-induced indirect toxicity. In addition, sinomenine inhibits amyloid beta-induced production of ROS, NO, and inflammation-related molecules in human astrocytes. Furthermore, this compound protects human neurons from amyloid beta-induced indirect toxicity.

## Materials and methods

### Preparation of oligomeric amyloid beta

The experimental procedures described previously [18] were used in this study with minor modifications. For preparation of oligomeric amyloid-beta, referred to as amyloid beta-derived diffusible ligands (ADDL), the amyloid beta (1–42) peptide (Bachem) was dissolved in 1,1,1,3,3,3-Hexafluoro-2-propanol (Fluka). The solution was aliquoted and dried in fume hood. The dried aliquots were stored at − 80 °C until use. The dried peptide was dissolved in dimethyl sulfoxide (DMSO; Sigma-Aldrich) at 5 mM concentration and further diluted with phosphate-buffered saline (PBS) to obtain a concentration of 100 μM of amyloid beta, and incubated for 24 h at 4 °C. The ADDL preparation was aliquoted and stored at − 80 °C until use.

### Cell culture and treatments

The mouse C8D1A astrocytic cell line, which has been used in previous studies [19, 20] was obtained from Dr. Anirban Basu of our institute. The hippocampal HT22 cells were a kind gift from Dr. D. Schubert, The Salk Institute, La Jolla, California. The preparation and characterization of primary human astrocytes and human neurons from aborted human fetuses has been described previously [21]. The guidelines of Institutional Human Ethics Committee of National Brain Research Centre, India, and the guidelines of Indian Council of Medical Research and Department of Biotechnology, India for Stem Cell Research were followed for use of the human tissue. Briefly, neural precursor cells were cultured in Neurobasal medium which was supplemented with basic fibroblast growth factor (bFGF), epidermal growth factor (EGF), neural cell survival factor-1, and N2 supplement. Neuronal differentiation was carried out in the neuronal medium which lacked bFGF and EGF, but contained brain-derived neurotrophic factor and platelet-derived growth factor-AB. Differentiation of neural precursor cells into astrocytes was carried out in Minimum Essential Medium containing 10% fetal bovine serum.

The C8D1A and hippocampal HT22 cells were cultured in Dulbecco’s Modified Eagle Medium, supplemented with 10% fetal bovine serum (Gibco), sodium bicarbonate (3.7 g/L, Sigma-Aldrich), and 1% penicillin and streptomycin solution (Gibco). The cells were serum starved for 2 h before treatment. To examine the effect of ADDL on the levels of ROS, the cells were treated with ADDL (0.5 μM) for 4 h. As a positive control for ROS generation, the cells were treated with LPS (10 μg/ml; Sigma-Aldrich) for 4 h. To examine the effects of ADDL on NO production, the cells were treated with ADDL (0.5 μM) for 3 h. To assess the levels of inflammation-related molecules, the cells were treated with ADDL (1.0 μM) for 6 h. For experiments examining the effects of sinomenine, after serum starvation, the cells were treated with sinomenine for 1.5 h before treatment with ADDL. Sinomenine was present during ADDL treatment also. Same procedure was followed for examining the effects of sinomenine on ROS, NO and inflammation-related molecules in human astrocytes. The stock solution (100 mM) of sinomenine (Sigma-Aldrich) was prepared in DMSO and used at a concentration of 100 μM. For collection of conditioned medium for indirect toxicity experiments using hippocampal neuronal cells, after serum starvation, the C8D1A cells were treated with sinomenine for 1.5 h followed by treatment with ADDL (1 μM) for 6 h, co-incident with ADDL treatment of a sister culture. After 3 washes, fresh culture medium without serum was added and incubation was carried out for a further 12 h period. The conditioned medium was collected and centrifuged to remove the cells. Same procedure was followed for collection of conditioned medium from human astrocytes for indirect toxicity experiments using human neurons. The HT22 cells were seeded in 8-well chambered slides (Nunc). After 24 h, the cells were serum-starved for 2 h and treated for 48 h with a mixture of 50% conditioned medium and 50% fresh culture medium without serum. Differentiated human neurons were seeded in the 8-well chambered slides, they were treated for 48 h with a mixture of 50% human astrocyte conditioned medium and 50% fresh neuronal medium.

### Reactive oxygen species assay

The level of intracellular ROS was measured using the reagent, 2′,7′-Dichlorofluorescin diacetate (DCFDA; Sigma-Aldrich). After treatment, the cells were washed and incubated for 1 h at 37 °C with DCFDA (5 μM) in culture medium without serum. The cells were washed with PBS and lysed in the lysis buffer (10 mM Tris pH 7.9, 150 mM NaCl, 1 mM ethylenediaminetetraacetic acid, 0.2 mM ethylene glycol-bis(β-aminoethyl ether)-N,N,N′,N′-tetraacetic acid, 0.2 mM Na_3_VO_4_, 0.5% NP-40 and 1% Triton X-100). After centrifugation, an aliquot (10 μl) of the supernatant was mixed with 90 μl of PBS in a 96-well black plate. Fluorescence was measured using Infinite M200 Pro Multimode Microplate Reader (Tecan) at an excitation wavelength of 485 nm and an emission wavelength of 530 nm. The ROS readings were normalized with the amount of protein present in each sample. Protein concentration was determined using bicinchoninic acid reagent (Thermo Fisher Scientific) with bovine serum albumin as standard.

### Nitric oxide assay

The level of NO was determined using the nitric oxide colorimetric assay kit (Biovision) according to the manufacturer’s guidelines. Briefly, after treatment, the culture medium was collected and centrifuged to remove cells. An aliquot (85 μl) of the supernatant was added to a well of a 96-well flat-bottom transparent plate. Subsequently, 5 μl each of nitrate reductase and enzyme cofactor was added to each well. The plate was covered and incubated at room temperature (RT) for 1 h. After this, 5 μl of enhancer was added to each well and incubated for 10 min. Finally, 50 μl each of Griess reagents R1 and R2 was added to each well and incubation was carried out for 10 min at RT. The absorbance was measured at 540 nm using Infinite M200 Pro Multimode Microplate Reader (Tecan).

### Cytometric bead array assay

The levels of inflammation-related molecules in C8D1A cells and human astrocytes were measured in cell-free culture medium using Cytometric Bead Array Mouse Inflammation Kit and Cytometric Bead Array Human Inflammation Kit, respectively (BD Biosciences). An aliquot (50 μl) of cell-free culture medium was mixed with 50 μl of bead mix and 50 μl of phycoerythrin detection reagent, and incubated at RT in the dark for 2 h (C8D1A samples) or 3 h (human astrocyte samples). After washing the beads with the wash buffer (provided with the kit), they were re-suspended in 300 μl of the wash buffer and analyzed in FACS Verse System using FCAP Array Software (BD Biosciences). In case of C8D1A cells, in one experiment, no signal was detected for TNF in any of the groups. In another experiment, no signal for TNF was detected in ADDL + sinomenine and sinomenine groups. In these experiments, the TNF readings were excluded from the analysis.

### Terminal deoxynucleotidyl transferase dUTP nick end labeling assay

The HT22 cells were treated with the conditioned medium obtained from C8D1A cells treated with ADDL in absence or presence of sinomenine. Similarly, human neurons were treated with the conditioned medium obtained from human astrocytes treated with ADDL in the absence or presence of sinomenine. After treatment, the cells were fixed and processed for Terminal deoxynucleotidyl transferase dUTP nick end labeling assay (TUNEL) assay using an in situ Cell Death Detection Kit, TMR red (Roche Diagnostics). After staining, the cells were mounted with the Vecta shield mounting medium containing 4′, 6-diamidino-2-phenylindole (DAPI; Vector Laboratories). The cells were imaged using Apotome fluorescence microscope (Carl Zeiss). The number of DAPI-stained and TUNEL-positive cells in 10–11 different frames were counted in each condition.

### Data analysis

Data were analyzed using repeated measures analysis of variance followed by Fisher’s least significant difference as a post hoc test. Differences were considered significant when the *p* value was less than 0.05. Data are expressed as mean ± standard error of mean.

## Results

### Sinomenine inhibits amyloid beta-induced increase in the level of reactive oxygen species in astrocytic cells

**Fig. 1:**
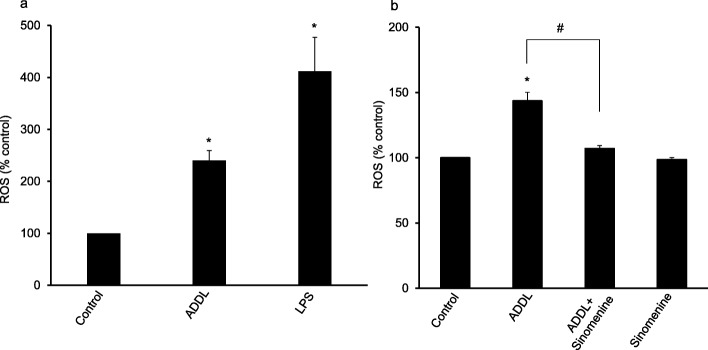
Sinomenine inhibits oligomeric amyloid beta-induced production of reactive oxygen species in astrocytic cells. The level of reactive oxygen species (ROS) was examined using the DCFDA reagent. **a** The oligomeric amyloid beta (ADDL) increases the level of ROS in astrocytic cells. The C8D1A astrocytic cells were treated with lipopolysaccharide (LPS) or ADDL and the levels of ROS were assessed. LPS and ADDL significantly increased the level of ROS in the cells (*n* = 3). **b** Sinomenine inhibits ADDL-induced increase in the level of ROS in astrocytic cells. The C8D1A astrocytic cells were treated with ADDL in the absence or presence of sinomenine and the levels of ROS were assessed. ROS production was increased in the ADDL-treated astrocytic cells. However, sinomenine inhibited ADDL-induced increase in ROS production. Sinomenine did not affect the level of ROS in the absence of ADDL treatment (*n* = 4 in all groups). Asterisk indicates significant difference from control (*p* < 0.05). # indicates significant difference (*p* < 0.05) between groups as indicated

Previous studies have shown that amyloid beta can affect the properties of astrocytes. Treatment of astrocytes with amyloid beta enhances the production of ROS in the cells [22, 23]. Thus, we first examined the level of ROS in the ADDL-treated astrocytic cells, C8D1A. As a positive control, we used LPS, which has been shown to increase ROS levels in astrocytes [24]. The ROS level was assessed using the commonly used reagent, DCFDA [18, 25]. Exposure of astrocytic cell to LPS or ADDL significantly increased ROS level (Fig. 1a). This result is consistent with previous reports which show that amyloid beta enhances production of ROS in astrocytes.

We next asked whether sinomenine affects the level of ROS in ADDL-treated astrocytic cells. The cells were treated with ADDL in the absence or presence of sinomenine and the level of ROS was examined. As expected, ADDL treatment of C8D1A cells enhanced the production of ROS. However, sinomenine inhibited the increase in the level of ROS in the ADDL-treated cells (Fig. 1b). Sinomenine alone had no significant effect on the ROS level in the cells. Thus sinomenine inhibits ADDL-induced increase in the production of ROS in astrocytic cells.

### Sinomenine inhibits amyloid beta-induced increase in nitric oxide level in astrocytic cells

**Fig. 2:**
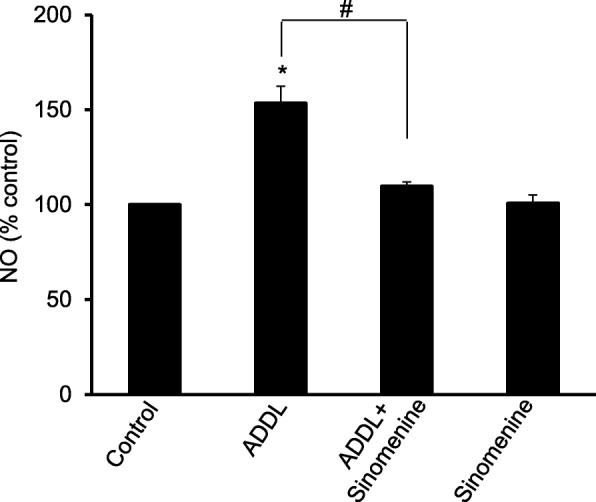
ADDL-induced increase in nitric oxide level in astrocytic cells is inhibited by sinomenine. The C8D1A astrocytic cells were treated with oligomeric amyloid beta (ADDL) in the absence or presence of sinomenine and the levels of nitric oxide (NO) were assessed. ADDL treatment increased NO level in the cells. However, sinomenine inhibited the ADDL-induced increase in the NO level (*n* = 4). Sinomenine did not affect NO level in the cells in the absence of ADDL treatment. Asterisk indicates significant difference from control (*p* < 0.05). # indicates significant difference (*p* < 0.05) between groups as indicated

Previous studies have shown that amyloid beta enhances the expression of inducible nitric oxide synthase (iNOS) and NO production in astrocytes [23, 26]. Thus, we asked whether sinomenine has any effect on amyloid beta-induced increase in the level of NO in astrocytes. The C8D1A cells were treated with ADDL in the absence or presence of sinomenine, and the level of NO was determined using commercially available kit. We observed that exposure of C8D1A cells to ADDL increased the level of NO (Fig. 2). However, sinomenine inhibited ADDL-induced increase in the level of NO. Sinomenine did not affect the level of NO in C8D1A cells in the absence of ADDL treatment. Thus, Sinomenine inhibits amyloid beta-induced increase in NO production in astrocytic cells.

### Sinomenine inhibits amyloid beta-induced increase in the levels of inflammation-related molecules in astrocytic cells

**Fig. 3:**
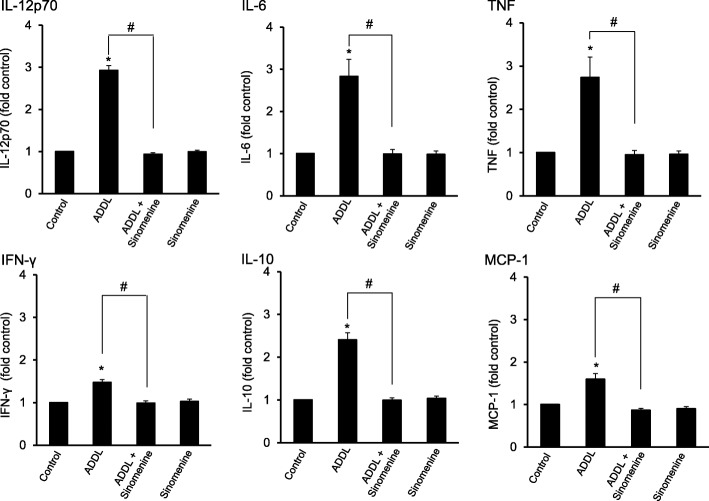
Sinomenine inhibits oligomeric amyloid beta-induced production of inflammation-related molecules in the astrocytic cells. The C8D1A astrocytic cells were treated with oligomeric amyloid beta (ADDL) in the absence or presence of sinomenine. The levels of inflammation-related molecules were assessed using cytometric bead array inflammation kit. Significant increase in the inflammation-related molecules was observed in the cells exposed to ADDL. However, sinomenine inhibited ADDL-induced increase in the levels of these molecules (IL-12p70, IL-6, IFN-γ, IL-10, MCP-1, *n* = 7; TNF, *n* = 5, in all groups). Sinomenine alone did not affect the levels of these molecules. Asterisks denote significant difference from control (*p* < 0.05). # indicates significant difference (*p* < 0.05) between groups as indicated

It has been shown previously that amyloid beta treatment of astrocytes increases the levels of inflammatory cytokines [27]. Here we examined whether sinomenine affects the production of inflammation-related molecules from ADDL-treated astrocytic cells. The C8D1A cells were treated with ADDL in the absence or presence of sinomenine, and cell-free culture medium was used to examine the levels of inflammation-related molecules. The culture medium from ADDL-treated astrocytic cells showed marked increase in the levels of interleukin (IL)-12p70, IL-6, tumor necrosis factor (TNF), interferon-γ (IFN- γ), IL-10 and monocyte chemoattractant protein-1 (MCP-1) (Fig. 3). Importantly, sinomenine inhibited the ADDL-induced increase in the levels of these molecules. Sinomenine did not affect the levels of these molecules in the astrocytic cells that were not treated with ADDL. Thus, sinomenine has inhibitory effect on ADDL-induced production of inflammation-related molecules in the astrocytic cells.

### Sinomenine protects hippocampal neuronal cells from indirect toxicity

**Fig. 4:**
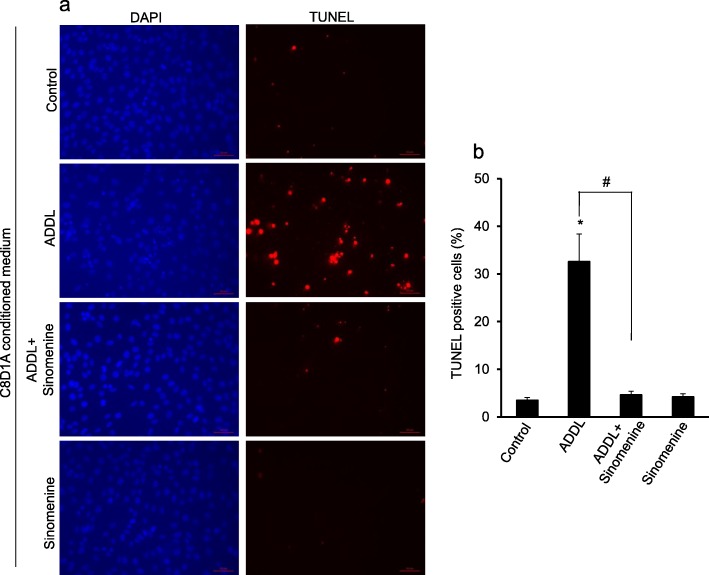
Sinomenine protects hippocampal neuronal cells from indirect toxicity. The C8D1A astrocytic cells were treated with oligomeric amyloid beta (ADDL) in the absence or presence of sinomenine. The conditioned medium was used to treat HT22 cells, and cell death was determined by TUNEL assay. The representative images of DAPI- or TUNEL-stained HT22 cells (**a**) and quantified summary data (**b**; n = 4) demonstrate that HT22 cells treated with the conditioned medium from ADDL-treated astrocytic cells showed increased number of TUNEL positive cells. However, sinomenine inhibited the ADDL-induced increase in the number of TUNEL positive cells. The HT22 cells treated with the conditioned medium from astrocytic cells that were treated with sinomenine alone, did not show increased number of TUNEL positive cells. Asterisk indicates significant difference from control (*p* < 0.05). # indicates significant difference (*p* < 0.05) between groups as indicated. Scale bar, 50 μm

Studies have shown that activated astrocytes release toxic molecules that cause neuronal damage. Considering our results on inhibitory effects of sinomenine on production of ROS and other factors from ADDL-treated astrocytic cells, we next asked whether this compound has any effect on indirect toxicity to hippocampal neuronal cells, HT22. These cells have previously been used for toxicity studies [18, 28]. We used TUNEL assay to determine cell death in HT22 cells. This assay involves labelling of DNA ends after it is fragmented during cell death. The C8D1A cells were treated with ADDL in the absence or presence of sinomenine. The conditioned medium from these cells was used to treat HT22 cells. We observed significant cell death reflected in an increase in the number of TUNEL**-**positive HT22 cells that were exposed to ADDL-treated C8D1A conditioned medium (Fig. 4). However, when the HT22 cells were exposed to the conditioned medium from C8D1A cells that were treated with ADDL in the presence of sinomenine, there was a marked decrease in the number of TUNEL-positive cells compared to the HT22 cells exposed to the condition medium from ADDL-treated C8D1A cells. The HT22 cells treated with the conditioned medium from sinomenine alone treated C8D1A cells did not show increased number of TUNEL positive cells. These results show that the conditioned medium from ADDL-treated astrocytic cells contains factors that cause toxicity to HT22 cells, and sinomenine protects these cells from indirect toxicity induced by ADDL acting on the astrocytic cells.

### Sinomenine inhibits amyloid beta-induced increase in the levels of reactive oxygen species and nitric oxide from human astrocytes

**Fig. 5:**
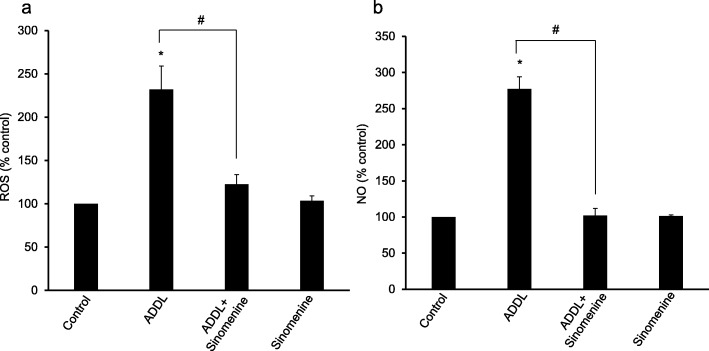
Sinomenine inhibits oligomeric amyloid beta-induced production of reactive oxygen species and nitric oxide in human astrocytes. The human astrocytes were treated with oligomeric amyloid beta (ADDL) in the absence or presence of sinomenine, and the levels of reactive oxygen species (ROS) and nitric oxide (NO) were assayed. **a** Effect of sinomenine on ADDL-induced increase in ROS level. The astrocytes treated with ADDL showed higher level of ROS. Sinomenine inhibited ADDL-induced increase in the level of ROS (*n* = 3). Sinomenine did not affect the level of ROS in the cells that were not treated with ADDL. **b** Effect of sinomenine on ADDL-induced production of NO. Treatment of astrocytes with ADDL led to a significant increase in the level of NO, whereas sinomenine inhibited ADDL-induced increase in NO level (n = 3). Sinomenine had no significant effect on NO level in the absence of ADDL treatment. Asterisks indicate significant difference from control (*p* < 0.05). # indicates significant difference (*p* < 0.05) between groups as indicated

After demonstrating that sinomenine inhibits oligomeric amyloid beta-induced production of ROS and NO in C8D1A astrocytic cells, we assessed these parameters in human primary astrocytes. The astrocytes were treated with oligomeric amyloid beta in the absence or presence of sinomenine and the levels of ROS were examined. Treatment of astrocytes with ADDL increased the level of ROS, but sinomenine inhibited ADDL-induced increase in the level of ROS (Fig. 5a). Sinomenine had no significant effect on the basal ROS level in the cells.

Next, we examined the effects of sinomenine on NO level in ADDL-treated human astrocytes. Exposure of astrocytes to ADDL increased the level of NO, but sinomenine showed inhibitory effect on ADDL-induced increase in NO level (Fig. 5b). Similar to a lack of effect on basal ROS level, sinomenine did not affect NO level in the absence of ADDL treatment. Collectively, the results show that sinomenine inhibits ADDL-induced production of ROS and NO in human astrocytes.

### Inhibitory effect of sinomenine on amyloid beta-induced increase in the levels of inflammation-related molecules in human astrocytes

**Fig. 6:**
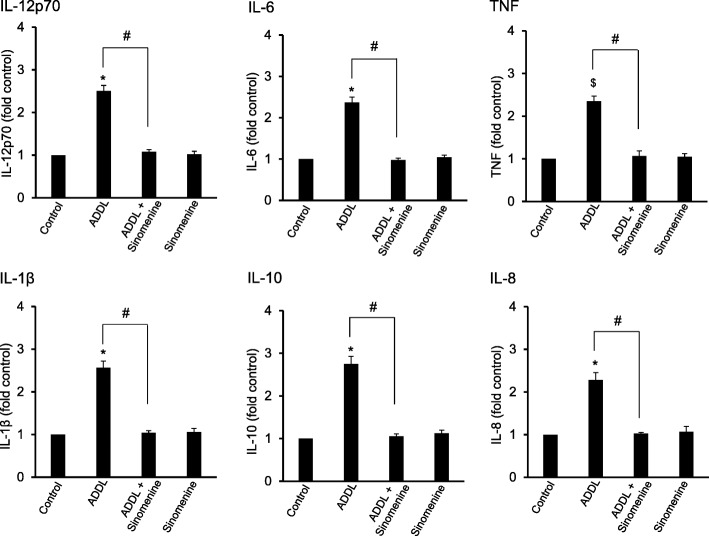
Sinomenine inhibits oligomeric amyloid beta-induced production of inflammation-related molecules in human astrocytes. The human astrocytes were treated with oligomeric amyloid beta (ADDL) in the absence or presence of sinomenine, and the levels of inflammation-related molecules were assessed. Treatment of astrocytes with ADDL led to an increase in the levels of inflammation-related molecules. Sinomenine inhibited ADDL-induced increase in the levels of these molecules (*n* = 6). Sinomenine alone did not affect the basal level of these molecules. Asterisks denote significant difference (*p* < 0.05) and $ indicates *p* < 0.057 from control. # indicates significant difference (*p* < 0.05) between groups as indicated

Since sinomenine showed inhibitory effects on oligomeric amyloid beta-induced increase in the levels of inflammation-related molecules in C8D1A cells, we asked whether this compound has any effect on these molecules in human astrocytes. The astrocytes were treated with ADDL in the absence or presence of sinomenine and the levels of inflammation-related molecules were examined. We found that whereas ADDL treatment of the astrocytes increased the levels of IL-12p70, IL-10, IL-6, IL-1β and IL-8, sinomenine inhibited ADDL-induced increase in the level of these molecules (Fig. 6). ADDL treatment increased the level of TNF, although it did not approach statistical significance (*p* < 0.057). Sinomenine inhibited ADDL-induced increase in TNF level. Sinomenine did not affect the levels of these molecules without ADDL treatment.

### Sinomenine protects human neurons from indirect toxicity

**Fig. 7:**
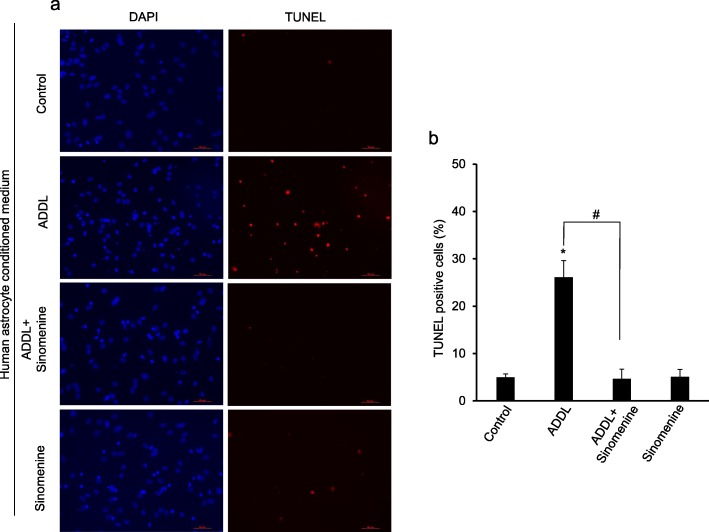
Sinomenine protects human neurons from indirect toxicity. The human astrocytes were treated with oligomeric amyloid beta (ADDL) in the absence or presence of sinomenine. The human neurons were treated with the conditioned medium from astrocytes and TUNEL assay was used to determine cell death. The representative images of DAPI- or TUNEL-stained neurons are shown in **a** and quantified summary data (*n* = 4) are shown in **b**. The neurons treated with the conditioned medium from ADDL-treated astrocytes showed increased number of TUNEL positive cells. However, ADDL-induced increase in the number of TUNEL positive cells was inhibited by sinomenine. The neurons exposed to the conditioned medium from astrocytes treated with sinomenine alone, did not show increased number of TUNEL positive cells. Asterisk indicates significant difference from control (*p* < 0.05). # indicates significant difference (*p* < 0.05) between groups as indicated. Scale bar, 50 μm

The earlier section shows that sinomenine confers protection to hippocampal neuronal cells from indirect toxicity induced by treatment of astrocytic cells with oligomeric amyloid beta. We next examined whether sinomenine has any effect on indirect toxicity to human neurons induced by treatment of human astrocytes with amyloid beta. The primary cultures of human astrocytes were treated with ADDL in the absence or presence of sinomenine. The conditioned medium was used to treat the primary cultures of human neurons. The human neurons that were exposed to the conditioned medium from ADDL-treated human astrocytes showed an increase in the number of TUNEL**-**positive cells indicating cell death (Fig. 7). However, compared to the neurons exposed to the conditioned medium from ADDL-treated astrocytes, the neurons exposed to the conditioned medium from astrocytes treated with ADDL in the presence of sinomenine, showed a significant reduction in the number of TUNEL-positive cells. Exposure of neurons to the conditioned medium from astrocytes treated with sinomenine alone did not increase the number of TUNEL positive cells. Collectively, the results show that sinomenine protects not only hippocampal neuronal cells from indirect toxicity induced by treatment of C8D1A cells with ADDL, but also protects human neurons from indirect toxicity induced by treatment of human astrocytes with amyloid beta.

## Discussion

The present study aimed to examine the effects of sinomenine, an alkaloid present in a Chinese medicinal plant, on amyloid beta-induced changes in astrocytes. Our results show that sinomenine inhibits the production of ROS, NO and inflammation-related molecules in amyloid beta-treated C8D1A cells and human astrocytes. Further, this compound inhibits indirect toxicity to hippocampal HT22 neuronal cells. Importantly, sinomenine inhibits indirect toxicity to human neurons.

The available evidence points to a crucial role of oligomeric form of amyloid beta in the development of AD. The brains of AD patients show neuronal cell death [9]. And, ADDL induces toxicity to neurons [29]. With respect to neurotoxicity, amyloid beta can kill neurons by directly acting on them. This mode of toxicity is referred to as direct neuronal toxicity. In the indirect mode of neurotoxicity, amyloid beta acts on the glial cells causing the release of toxic and inflammatory factors, which eventually kill neurons. Both toxicity mechanisms play important roles in the development of AD.

The astrocytes help in neuronal functions [30]. Consequently, dysfunction of astrocytes could severely affect neuronal function contributing to neurodegenerative diseases including AD. Reactive astrocytes have been observed in the brains of AD patients and in the brains of animal models of AD [9, 30]. Available evidence suggests that oxidative stress is an important contributing factor in the development of AD. AD brains show oxidative stress and higher levels of iNOS [5, 9, 31]. In addition, the brains of AD patients show enhanced levels of inflammatory molecules [5]. In vitro studies also have shown that amyloid beta induces the production of ROS, NO and inflammatory molecules in astrocytes [26, 32–34]. Consistent with these observations, we found that ADDL-treated C8D1A cells and human astrocytes show higher levels of ROS, NO and inflammation-related molecules. Importantly, sinomenine inhibited ADDL-induced increase in the production of these factors. We found that ADDL enhanced the level of IL-10, an anti-inflammatory molecule. Increased level of IL-10 has been detected in AD brains [35]. Upregulation of IL-10 has been observed in AD transgenic animals [36]. Garwood and colleagues found a trend towards increase in IL-10 after treatment of astrocytes with amyloid beta, although the effect was not significant [37]. It is unclear why ADDL enhanced the level of IL-10, an anti-inflammatory molecule. One possibility is that the cells try to limit inflammation by increasing the level of anti-inflammatory molecule, but inflammation prevails.

Our study shows that sinomenine inhibits oligomeric amyloid beta-induced production of ROS, NO and inflammation-related molecules from astrocytes. Since sinomenine has inhibitory effects on activation of nicotinamide adenine dinucleotide phosphate oxidase, iNOS expression and nuclear factor kappa B activation [14, 15], these properties of sinomenine may, at least in part, be involved in its effects on amyloid beta-induced production of toxic and inflammation-related molecules. Further studies are needed to fully elucidate the mechanisms involved in the effects of sinomenine on production of these molecules.

It has been shown that neurons are more sensitive to amyloid beta induced toxicity in the presence of astrocytes [38]. In addition, the conditioned medium from amyloid beta-treated astrocytes causes oxidative stress and cell death in neurons [27]. Our study also shows that the conditioned medium from amyloid beta-treated astrocytic cells causes toxicity to hippocampal neuronal cells. However, the conditioned medium from sinomenine and amyloid beta-treated astrocytic cells does not lead to enhanced neurotoxicity. We extended the study to human astrocytes and neurons. The conditioned medium from amyloid beta-treated human astrocytes causes toxicity to neurons. However, the conditioned medium from sinomenine and amyloid beta-treated astrocytes does not lead to enhanced neurotoxicity. Thus, sinomenine protects HT22 cells and human neurons from indirect toxicity induced by amyloid beta. Previous studies have shown that sinomenine is able to cross blood brain barrier [11, 39, 40]. Considering the findings of this study combined with the results of our previous study, which showed that sinomenine inhibits microglial activation and indirect toxicity induced by amyloid beta [18], it would be interesting to explore sinomenine as a potential therapeutic compound in animal models of AD.

## Data Availability

The datasets used and/or analysed during the current study are available from the corresponding author on reasonable request.

## Abbreviations

AD: Alzheimer’s disease
ADDL: Amyloid beta-derived diffusible ligands
bFGF: Basic fibroblast growth factor
DAPI: 4′,6-diamidino-2-phenylindole
DCFDA: 2′,7′-Dichlorofluorescin diacetate
DMSO: Dimethyl sulfoxide
DNA: Deoxyribonucleic acid
dUTP: Deoxyuridine triphosphate
EGF: Epidermal growth factor
IFN-γ: Interferon-γ
IL-10: Interleukin-10
IL-12p70: Interleukin-12p70
IL-1β: Interleukin-1 beta
IL-6: Interleukin-6
IL-8: Interleukin-8
iNOS: Inducible nitric oxide synthase
LPS: Lipopolysaccharide
MCP-1: Monocyte chemoattractant protein-1
NO: Nitric oxide
PBS: Phosphate buffered saline
ROS: Reactive oxygen species
RT: Room temperature
TNF: Tumor necrosis factor
TUNEL: Terminal deoxynucleotidyl transferase-mediated dUTP nick-end labeling
